# Prediction of Solvent Composition for Absorption-Based Acid Gas Removal Unit on Gas Sweetening Process

**DOI:** 10.3390/molecules29194591

**Published:** 2024-09-27

**Authors:** Mochammad Faqih, Madiah Binti Omar, Rafi Jusar Wishnuwardana, Nurul Izni Binti Ismail, Muhammad Hasif Bin Mohd Zaid, Kishore Bingi

**Affiliations:** 1 Department of Chemical Engineering, Universiti Teknologi PETRONAS, Seri Iskandar 32610, Malaysia; madiah.omar@utp.edu.my (M.B.O.); rafi_24002236@utp.edu.my (R.J.W.); nurul_19001247@utp.edu.my (N.I.B.I.);; 2 Department of Electrical and Electronics Engineering, Universiti Teknologi PETRONAS, Seri Iskandar 32610, Malaysia; bingi.kishore@utp.edu.my

**Keywords:** solvent composition, acid gas removal unit, XGBoost, MDEA, PZ

## Abstract

The gas sweetening process is essential for removing harmful acid gases, such as hydrogen sulfide (H_2_S) and carbon dioxide (CO_2_), from natural gas before delivery to end-users. Consequently, chemical absorption-based acid gas removal units (AGRUs) are widely implemented due to their high efficiency and reliability. The most common solvent used in AGRU is monodiethanolamine (MDEA), often mixed with piperazine (PZ) as an additive to accelerate acid gas capture. The absorption performance, however, is significantly influenced by the solvent mixture composition. Despite this, solvent composition is often determined through trial and error in experiments or simulations, with limited studies focusing on predictive methods for optimizing solvent mixtures. Therefore, this paper aims to develop a predictive technique for determining optimal solvent compositions under varying sour gas conditions. An ensemble algorithm, Extreme Gradient Boosting (XGBoost), is selected to develop two predictive models. The first model predicts H_2_S and CO_2_ concentrations, while the second model predicts the MDEA and PZ compositions. The results demonstrate that XGBoost outperforms other algorithms in both models. It achieves R^2^ values above 0.99 in most scenarios, and the lowest RMSE and MAE values of less than 1, indicating robust and consistent predictions. The predicted acid gas concentrations and solvent compositions were further analyzed to study the effects of solvent composition on acid gas absorption across different scenarios. The proposed models offer valuable insights for optimizing solvent compositions to enhance AGRU performance in industrial applications.

## 1. Introduction

The gas sweetening process plays a crucial role in refinery or chemical industries to ensure the quality of gas products, such as natural gas. Although natural gas is known for its numerous benefits including environmentally friendliness, extensive accessibility, and high reliability, this gas inherently contains several acidic substances, particularly hydrogen sulfide (H_2_S) and carbon dioxide (CO_2_) [1]. These two acid gases need to be removed since they may cause various damages to the environment and human health [2]. Furthermore, they can damage the equipment and facility such as gas pipelines. Thus, the sour gas from the original source has to undergo the sweetening process to become sweet gas before being utilized. The term “sweet gas” refers to the purified gas which contains an acceptable quantity of H_2_S. Conversely, the gas with high H_2_S or the natural gas before being purified is referred to as “sour gas”. Industrially, the permitted concentration of H_2_S should be less than 4 to 20 ppm and below 3–4 mole% for CO_2_ [3].

Several techniques applied in the gas sweetening process include absorption with a chemical and physical solvent, membranes, adsorption, and the oxidation process. In acid gas removal unit (AGRU) technology, chemical absorption has been widely applied with alkanolamines as the solvent that is often used for commercial applications. Amines are mainly categorized into primary, secondary, and tertiary. The examples of chemical compounds for these three amines are diglycolamine amine (DGA), diethanolamine (DEA), and monodiethanolamine (MDEA), respectively. Among all types of amines, MDEA is the most commonly used amine solvent due to its lower degradation and corrosion rates. However, MDEA has a lower absorption rate of CO_2_. This is due to a slow formation of bicarbonate when this solvent reacts with CO_2_. Consequently, the use of additives or an amine mixture is recommended to overcome the limitation of MDEA.

Amine mixtures aim to leverage the strengths of each amine by compensating for the limitation of the single amines to improve the absorption rate. Some additives that are usually blended with MDEA include piperazine (PZ), Triethylamine (TEA), sulfolane, and DEA. Industrially, the MDEA concentration ranges from 30 to 45 wt%, and additional activator can be kept around 5 to 20 wt%. Among the additives, MDEA is commonly mixed with PZ which has a high reaction rate towards CO_2_ [4,5]. Even though the amine mixture exhibits a significant improvement in absorption effectiveness, the removal efficiency is affected by several factors [6,7]. These factors include the temperature, pressure, and flowrate of the sour gas and solvent. Apart from that, the mixture composition of the solvent blend is one crucial factor for optimizing the removal efficiency.

The conventional method of solvent optimization mainly relies on experiment and simulation by randomly variegating the composition of the solvent mixture. Umer in [8] simulated the blended DGA and MDEA using Hysys to observe the energy consumption in the acid gas removal process. The result exhibits that the addition of 0–15 wt% DGA to MDEA significantly improves the operational energy savings. Farooqi in [9] used a mixture of diisopropanolamine (DIPA) and TEA to achieve an optimized absorption rate of H_2_S and CO_2_. TEA (35 to 50 wt%) acts as the base amine and DIPA (0–15 wt%) is used as the additive amine with the variation in pressure ranging from 10 to 18 bar. The study shows that there was a significant impact aligning with the increase in operating pressure. Different from the mentioned studies, Law in [10] combined MEA and MDEA to simulate acid gas removal. The simulation exhibits that a 5.5% efficiency improvement with 91.27% removal rate of CO_2_ was achieved. AGRU optimization mainly simulated in Hysys software 14.0 which has various useful packages to model gas sweetening plants. Instead of only simulating the acid gases’ capture, Hamid et al. in [11] experimentally observed the use of MEA with different compositions for CO_2_ removal. The MEA with a concentration ranging from 25 to 33 wt% was applied in a laboratory pilot plant. However, it faced some limitations such as being time-consuming and resource-intensive. To this end, several attempts at employing data-driven techniques were proposed by researchers.

Hakimi in [12] developed a predictive model for H_2_S concentration using Artificial Neural Networks (ANNs) in comparison with Multi Linear Regression (MLR). The model was trained using simulated data with solvent blending of MDEA and PZ. The model showed an outstanding performance represented by an R-squared of 0.96. A similar attempt at employing an ANN in AGRU was proposed by Alardhi [13] and Salooki [14]. Both researchers utilized actual data from a gas sweetening plant with a single solvent. The predictive performance of R-squared lies between 0.9 and 0.92, respectively. Another attempt was proposed by Adib [15] and Rahaei [16] to predict H_2_S using the Support Vector Machine (SVM). SVM was powerful in predicting the H_2_S concentration with an R-squared of 0.97 in [16]. However, SVM is less accurate when being compared with Random Forest as reported in [15]. A recent study of H_2_S and CO_2_ prediction was initiated using Extreme Gradient Boosting (XGBoost). As reported in [17], Yogesh implement different solvents include MDEA + PZ, DEA, and MDEA. The result exhibits that the highest accuracy was achieved by the single solvent of DEA. In contrast, XGBoost results in a low accuracy compared with linear regression for MDEA + PZ.

Ultimately, the solvent optimization techniques available in the literature generally focus on predicting the concentration of acid gases (H_2_S and CO_2_) by arbitrary determining the composition of single or blended amines. There has been limited study on predicting the optimum composition of a solvent mixture as a strategy to optimize the absorption rate on AGRU. Therefore, this paper aims to develop a novel predictive model to predict the solvent composition. The combination of MDEA + PZ with variation in sour gas composition will be observed in this study. XGBoost is selected for developing the model due to its fast computation, superior accuracy, and advanced regularization capability to handle overfitting. The proposed model will be further optimized and compared with other comprehensive algorithms as a benchmark. The proposed model contributes to the ease of the operator or stakeholders in a gas sweetening plant in determining the suitable solvent composition. Hence, it will help to optimize their acid gas removal process.

## 2. Results and Discussion

In this section, the result of two models is presented numerically and graphically. All performance metrics are deeply discussed to measure the accuracy of the model in predicting the desired variables. The discussion begins with the correlation test result. Then, the performance of the predictive model of H_2_S and CO_2_ concentration is evaluated. Subsequently, the result of solvent composition prediction is presented. The proposed model is further compared with other comprehensive algorithms such as Linear Regression (LR), k-Nearest Neighbors (kNN), and Support Vector Machine (SVM).

### 2.1. Correlation Analysis

**Figure 1:**
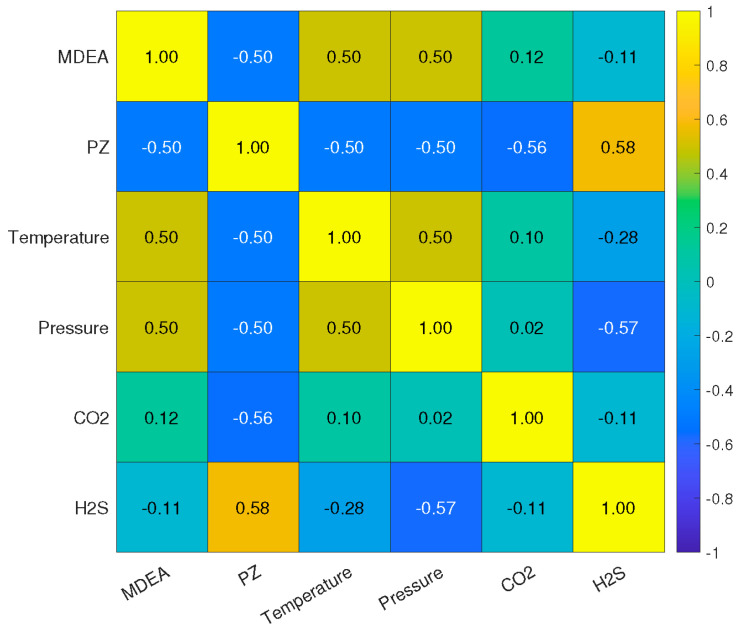
Correlation test heatmap.

The correlation test result is mapped in Figure 1. MDEA and PZ are strongly correlated with a negative coefficient of −0.50, showing an inverse relationship between these two solvents. This means when the concentration of MDEA increases, the concentration of PZ decreases, and vice versa. This inverse relationship indicates that the solvents may be substitutable to some extent depending on the specific needs for CO_2_ and H_2_S removal. Both temperature and pressure exhibit a positive correlation of 0.50 with MDEA and a negative coefficient of −0.50 with PZ. This indicates that increases in temperature and pressure are associated with proportional increases in the concentrations of MDEA. Conversely, the concentration of PZ decreases when temperature and pressure increase, and vice versa. Such a relationship suggests that operational conditions like temperature and pressure can be manipulated to optimize the solvent concentrations for better absorption efficiencies.

CO_2_ has a slightly positive correlation with MDEA (0.12) but a negative correlation with PZ (−0.56). This suggests that MDEA may slightly increase the presence of CO_2_ in the sweet gas, whereas PZ helps to decrease its concentration. This relationship can be explained by the drawback of MDEA which does not directly react with CO_2_. Thus, the addition of PZ improves the absorption rate of CO_2_. To this end, the strategic adjustment of PZ levels could be key in managing CO_2_ concentrations. H_2_S exhibits a weak negative correlation with MDEA (−0.11) and a notably strong positive correlation with PZ (0.58). This indicates that the increase in MDEA will capture the concentration of H_2_S more effectively. On the other hand, the higher levels of PZ correlate with higher concentrations of H_2_S in the sweet gas. Therefore, careful management of PZ levels is crucial for controlling H_2_S concentrations effectively.

As mentioned previously, the data used in this paper are generated with five different sour gas compositions. The variety of sour gas composition leads to distinct H_2_S and CO_2_ behavior and its absorption capability over the solvent composition. Table 1 presents the statistics of the acid gases contained in sweet gas for each scenario.

Across five distinct scenarios of sour gas composition, Scenario 4 shows the highest H_2_S and CO_2_ concentrations with peak amounts of 121.8 ppm and 8.44%, respectively. For H_2_S, the next highest concentration is gained by Scenario 1 (104.2 ppm) and Scenario 3 (101.2 ppm). This order of H_2_S concentration amount in sweet gas highly relates with the composition of its molar fraction in sour gas, as mentioned in Table 1. The highest CO_2_ concentration is found in Scenario 4, followed by Scenario 5 (7.23%) and Scenario 1 (5.97%). Similarly, the concentration contained in sweet gas is highly influenced by the composition of its molar fraction in sour gas.

In Scenario 1, H_2_S exhibits substantial variability, ranging from 35.98 ppm to 104.2 ppm with an average of 53.05 ppm. The CO_2_ levels, while relatively low across the board, range from 0.7303% to 5.975% with an average of 1.312%, indicating that CO_2_ is consistently removed efficiently. Moving to Scenario 2, the system shows a more stable concentration of H_2_S, with concentrations stretching from 12.05 ppm to 35.78 ppm and a modest average of 18.21 ppm. CO_2_ concentrations in this scenario also remain low, demonstrating that the solvents consistently absorb the CO_2_. Scenario 3 presents a median performance in H_2_S removal with concentrations peaking at 101.2 ppm and dipping to 31.71 ppm, while maintaining a moderate average of 43.06 ppm. This indicates a balance in solvent efficacy, particularly as CO_2_ levels remain particularly low, ranging from 0.4652% to 3.382%, with an average of 0.843%.

Lastly, Scenario 5 indicates moderate H_2_S capture capabilities, with concentrations ranging from 18.99 ppm to 66.62 ppm and averaging 33.90 ppm. CO_2_ removal is maintained effectively, with concentrations from 0.8473% to 7.239% and an average of 1.516%. These results underscore the necessity of finely tuning solvent compositions to specific gas compositions to optimize the gas sweetening process, as higher PZ concentrations generally correlate with increased H_2_S in the sweet gas while effectively reducing CO_2_ levels.

### 2.2. Predictive Model of H_2_S and CO_2_ Concentration

The performance metrics for the prediction of H_2_S and CO_2_ concentrations using various models are tabulated in Table 2. The result underscores the robustness and effectiveness of the proposed XGBoost model in comparison to other algorithms such as Linear Regression (LR), k-Nearest Neighbors (kNN), and Support Vector Machine (SVM).

For H_2_S concentration, the proposed XGBoost model consistently demonstrates superior performance across all scenarios in both the training and testing prediction. It achieves a near-perfect prediction accuracy with an R^2^ value approaching 1.0 and extremely low RMSE and MAE values. The highest performance is found in Scenario 2, where it reaches R^2^ = 0.998 during testing with an RMSE of 0.154 and MAE of 0.081. This indicates the minimal prediction error and high reliability of the model. Similarly, in predicting CO_2_ concentrations, XGBoost maintains an exemplary performance. It achieves R^2^ values again close to 1.0, and negligible RMSE and MAE values across all scenarios. The precision of XGBoost in the testing data for Scenario 3 is highlighted. It gained an R^2^ = 0.995, RMSE of 0.019, and MAE of 0.006, indicating highly accurate predictions.

In contrast, Linear Regression shows a significantly weaker performance, especially for H_2_S. This model struggles with lower R^2^ values and higher error metrics, such as in Scenario 4 where the R^2^ is only 0.415 with an RMSE of 7.549 and MAE of 5.89. For CO_2_, although its performance slightly improves, it remains suboptimal compared to XGBoost. On the other hand, the k-Nearest Neighbors algorithm performed better than LR, particularly for H_2_S with R^2^ values above 0.98 in most scenarios. However, it still does not match the near-perfect metrics of XGBoost. In CO_2_ predictions, kNN exhibits a good accuracy but falls short of the high benchmarks set by the proposed XGBoost model. Support Vector Machine demonstrates a moderate performance. For H_2_S, the R^2^ values are consistently lower than those of XGBoost, and the error rates are higher, as seen in Scenario 4 (RMSE of 2.597). In CO_2_ predictions, the performance of SVM is stable at above 0.87 but not as impressive as XGBoost.

To this end, the XGBoost model stands out as the most accurate model in predicting H_2_S and CO_2_ concentrations. It is affirmed by its consistently high R^2^ values and minimal error metrics across all tested scenarios. Its robustness and accuracy surpass the performance of traditional models.

**Figure 2:**
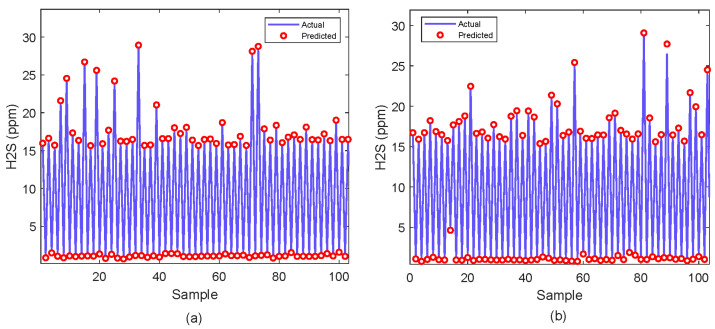
Prediction of H_2_S concentration; (**a**) Training sample (**b**) Testing sample.

**Figure 3:**
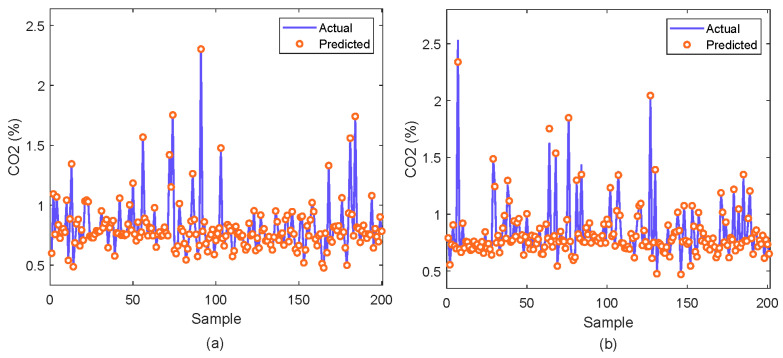
Prediction of CO_2_ concentration; (**a**) Training sample (**b**) Testing sample.

**Figure 4:**
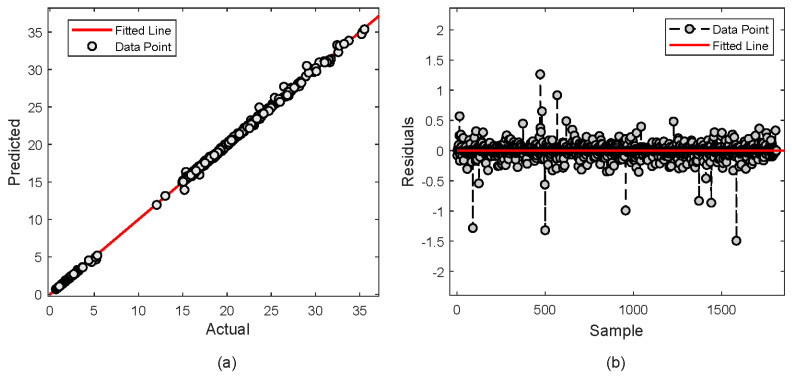
Actual vs. predicted values of H_2_S concentration; (**a**) Actual vs predicted plot (**b**) Residual error.

**Figure 5:**
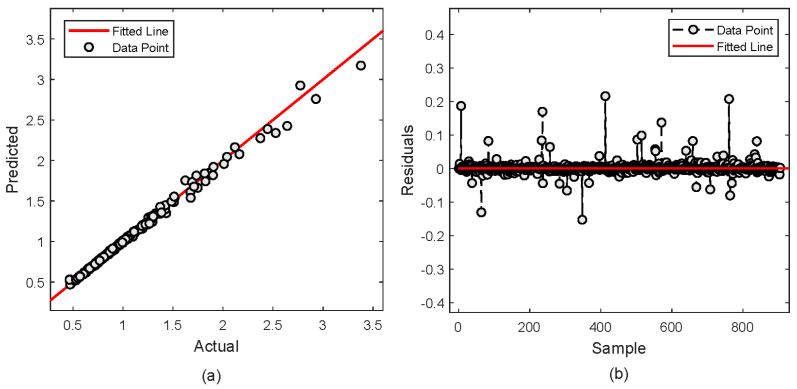
Actual vs. predicted values of CO_2_ concentration; (**a**) Actual vs predicted plot (**b**) Residual error.

The proposed XGBoost model is further analyzed graphically in Figure 2, Figure 3, Figure 4 and Figure 5. The prediction of H_2_S concentrations showcases its precision in both training and testing data as depicted in Figure 2a,b, respectively. In the training data, the prediction closely aligns with the actual values. It is indicated by the overlap of the predicted (red circles) and actual (blue lines) points across samples, with very few deviations. This indicates a high degree of accuracy and a good fit of the model to the training dataset. The testing data show a similar trend, where the predicted values mostly mirror the actual data. However, few discrepancies are noticeable in higher concentration ranges.

Similarly, for the CO_2_ predictions shown in Figure 3a,b, the model generally tracks the actual data closely in both the training and testing data. The predicted values (orange circles) capture the overall trend of CO_2_ concentrations (blue lines), though there are challenges in accurately predicting the peaks.

The analysis is further continued to comprehensively evaluate the performance through an analysis of the fit and residuals. Figure 4a depicts the actual over the predicted values. It is observed that the data points closely aligning with the fitted line, indicating excellent model fit and high consistency between the predictions and actual values. This alignment indicates the model captures the trend in H_2_S concentration accurately without significant bias. Figure 4b shows the residuals, the differences between predicted and actual values. The prediction is centered around zero and mostly contained within a narrow range, highlighting minor prediction errors.

Similarly, for CO_2_ as depicted in Figure 5a, the model predictions closely follow the actual data, also aligning well with the fitted line which reflects a strong model fit and accurate capture of the CO_2_ concentration trends. The residuals for CO_2_ presented in Figure 5b similarly show a distribution centered around zero, with the residuals predominantly lying within a narrow band. The absence of any discernible patterns or systematic errors in the residuals across all samples confirms the accuracy and reliability of the XGBoost model across varying levels of H_2_S concentration.

### 2.3. Predictive Model of Solvent Composition

The prediction result of the MDEA and PZ compositions is tabulated in Table 3. XGBoost exhibits an outstanding performance in both MDEA and PZ predictions across all scenarios.

For MDEA, the R^2^ of XGBoost for the training and testing data is exceptionally high with value approaching 1.0. The performance is supported by minimal RMSE and MAE values which range from 0.066 to 0.159 in training RMSE and as low as 0.045 to 0.107 in training MAE. Similarly, the RMSE values for the testing dataset range from 0.448 to 0.668 and MAE from 0.135 to 0.274, which indicates precise predictions.

Conversely, LR shows a significantly weaker performance for MDEA, with R^2^ values in training never exceeding 0.443 and testing R^2^ values not improving beyond 0.462. The associated RMSE and MAE are also high, with the training RMSE reaching up to 1.727 and an MAE up to 1.336 which underscores a poor fit.

For PZ predictions, XGBoost maintains near-perfect R^2^ scores, with training dataset values of 0.999 and testing values being equally robust. The error metrics remain impressively low, with training RMSE and MAE not exceeding 0.041 and 0.019, respectively. Similarly, the R^2^ of the testing values is equally low. On the other hand, kNN and SVM show variable but mostly more promising results than LR. kNN achieves strong R^2^ values in later scenarios for MDEA, such as 0.965 in training, and a similarly high performance for PZ with minimal deviation from perfect predictions.

SVM also shows competent performance, particularly in PZ predictions, with testing R^2^ values of 0.993 and an RMSE as low as 0.117, suggesting it can be a reliable model albeit not as consistently high-performing as XGBoost.

Following this, the proposed XGBoost model shows an outstanding capability to predict the solvent composition of MDEA and PZ. It shows that the solvent composition can be accurately predicted which will be useful for optimizing the absorption rate in future.

**Figure 6:**
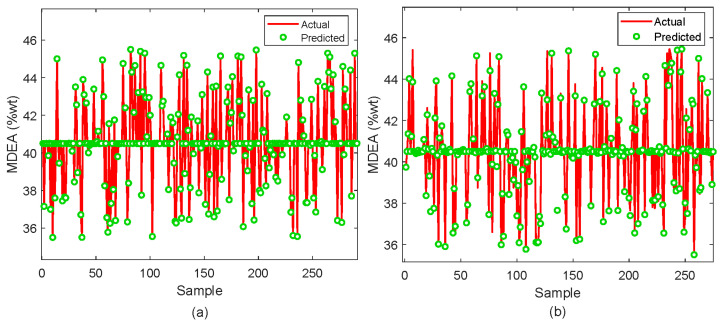
Prediction of MDEA composition; (**a**) Training sample (**b**) Testing sample.

**Figure 7:**
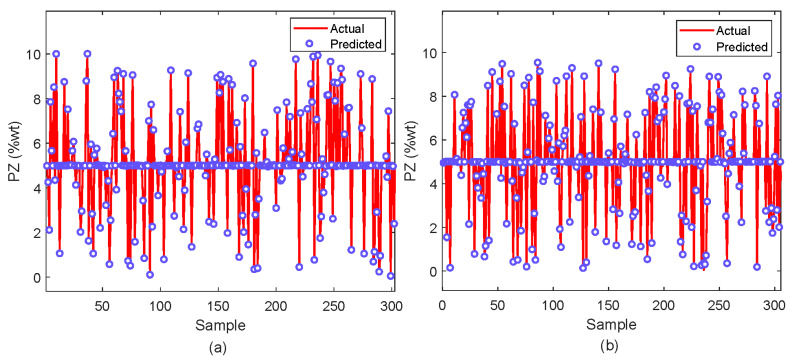
Prediction of PZ composition; (**a**) Training sample (**b**) Testing sample.

**Figure 8:**
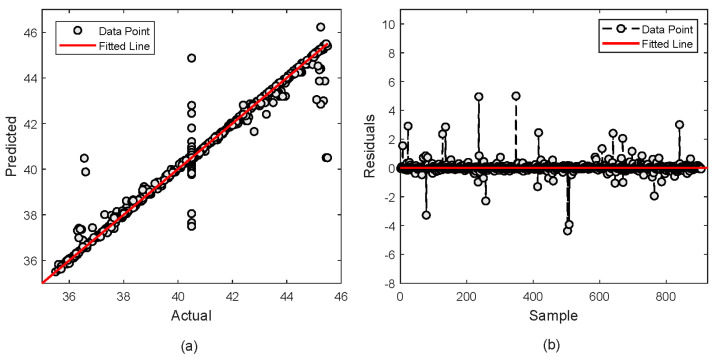
Actual vs. predicted values of MDEA composition; (**a**) Actual vs predicted plot (**b**) Residual error.

**Figure 9:**
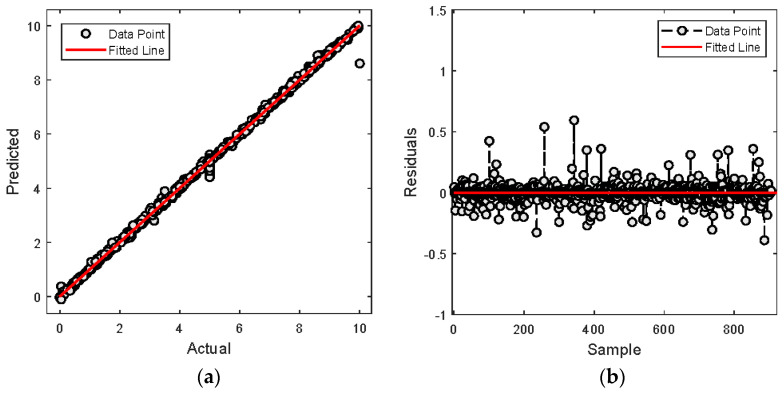
Actual vs. predicted values of PZ composition; (**a**) Actual vs predicted plot (**b**) Residual error.

The analysis for the XGBoost model is further continued through graphical evaluation as depicted in Figure 6, Figure 7, Figure 8 and Figure 9. As shown in Figure 6a for MDEA, the prediction on the training dataset perfectly aligns with the actual values. Similarly, the testing dataset in Figure 6b depicts an excellent prediction as represented by the predicted values (green circles) mostly following the actual values (red lines). However, there are some discrepancies noticeable at higher concentrations.

Similarly, for the PZ predictions shown in Figure 7a,b, the model mostly follows the actual data closely in both the training and testing data. Furthermore, the predicted values (blue circles) capture the overall trend of PZ concentrations (red lines).

A deeper analysis is further performed to evaluate the performance of the proposed XGBoost model by observing the fitness and residual plot. For MDEA, Figure 8a shows that most of prediction falls under the fitness line. However, some predictions deviate from the fitted line. It is observed that about 20 points are located away which indicates slight discrepancies in the model. Nevertheless, the closeness of most points to the fitted line demonstrates that predictions are very close to the actual values. The residual plot in Figure 8b also depicts less error as shown by most of the data points being distributed closely with the fitted line.

For PZ, Figure 9a demonstrates the predicted values precisely fit with the fitted line. It is observed that only one point has deviated from the line. The plot reflects a highly effective model with consistent predictive accuracy. In term of residuals in Figure 9b, most of the data are distributed closely with zero. It represents the model that accurately predicts the actual values with much less error.

### 2.4. Effect of Solvent Composition over H_2_S and CO_2_ Concentration in Sweet Gas

The predicted H_2_S and CO_2_ concentration along with the predicted MDEA and PZ composition from previous models are then used to observe the behavior of acid gas absorption for each scenario. The effect of solvent composition over the concentration of H_2_S and CO_2_ in sweet gas is evaluated through a contour plot.

**Figure 10:**
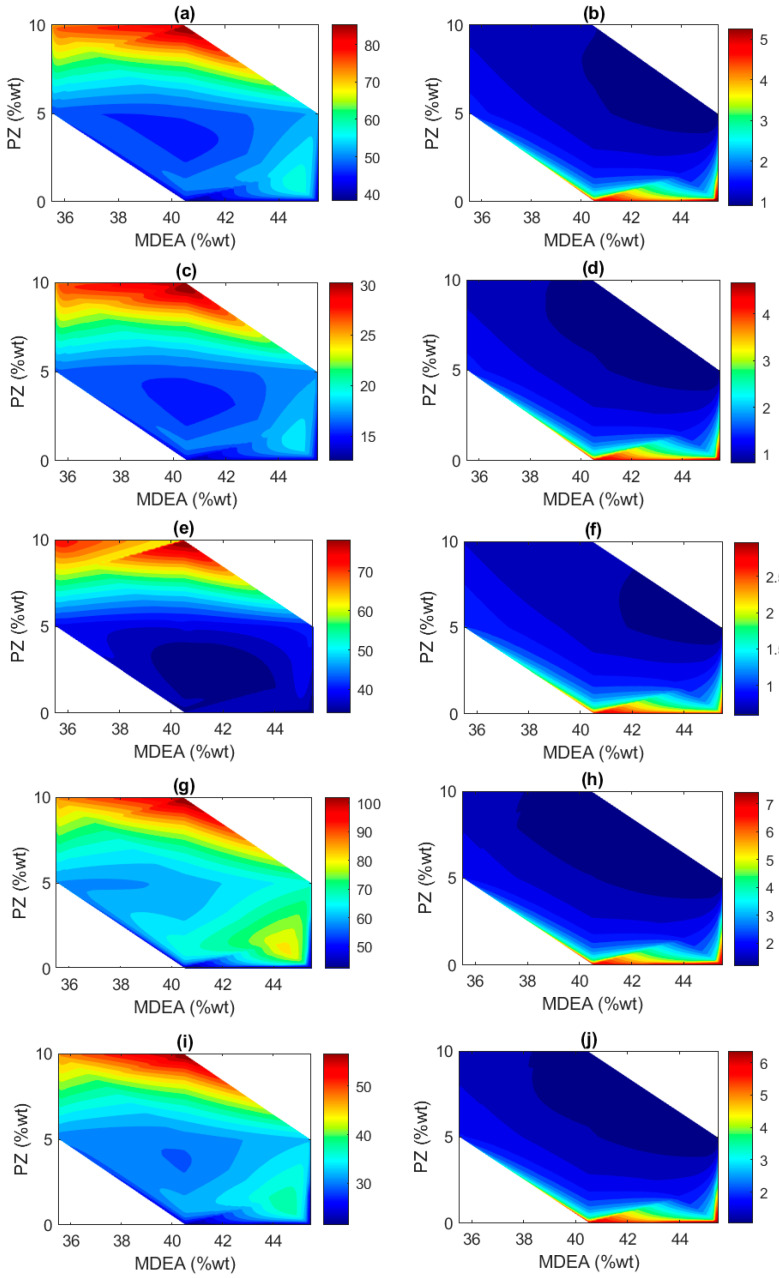
Predicted solvent composition against predicted acid gases in sweet gas for different sour gas compositions of (**a**) H_2_S in Scenario 1; (**c**) H_2_S in Scenario 2; (**e**) H_2_S in Scenario 3; (**g**) H_2_S in Scenario 4; (**i**) H_2_S in Scenario 5; and for (**b**) CO_2_ in Scenario 1; (**d**) CO_2_ in Scenario 2; (**f**) CO_2_ in Scenario 3; (**h**) CO_2_ in Scenario 4; (**j**) CO_2_ in Scenario 5.

Figure 10 depicts the distribution of the H_2_S and CO_2_ concentration in sweet gas over the composition of MDEA and PZ for each scenario. Lower concentrations, as represented by a darker blue hue, represent a better absorption performance.

In Scenario 1, the contour plot for H_2_S in Figure 10a shows high concentrations mostly occurring at higher percentages of PZ, as represented by a dark red color. This pattern suggests that increasing PZ does not effectively enhance H_2_S absorption, while a lower concentration is found at a high percentage of MDEA and low percentage of PZ, as shown by the dark blue color. Conversely, the CO_2_ plot in Figure 10b reveals higher concentrations primarily at higher MDEA levels with moderate PZ percentages, as represented by the dark red color at the bottom of the contour. This indicates that MDEA as single solvent might not suffice for efficient CO_2_ capture.

Scenario 2 exhibits a broad distribution of H_2_S concentrations across varying percentages of MDEA and PZ. Figure 10c shows relatively low concentrations of H_2_S across a range of MDEA and PZ percentages. This suggests that the solvent composition is quite effective at capturing H_2_S under this specific scenario of sour gas composition. The distribution being more spread out across different solvent compositions indicates that even at varied levels of MDEA and PZ, the system is capable of maintaining low H_2_S concentrations effectively. Figure 10d clearly shows that CO_2_ concentrations are lowest at higher percentages of MDEA and moderate percentages of PZ. This insight indicates that PZ enhances the kinetics of CO_2_ absorption.

In Scenario 3, the distribution of H_2_S concentrations in Figure 10e depicts that higher concentrations of MDEA coupled with relatively low levels of PZ optimize H_2_S absorption in sweet gas. This is shown by the significant region of low H_2_S concentration (deep blue area) spanning from approximately 40% to 44% MDEA with PZ levels up to 5%. The effectiveness in absorbing H_2_S decreases noticeably as the PZ concentration increases beyond this range, demonstrated by a steep transition to warmer colors indicating higher H_2_S concentrations. The plot distinctly exhibits that MDEA is the primary driver for H_2_S absorption efficiency in this scenario, with its performance notably diminished by increasing levels of PZ. The CO_2_ plot in Figure 10f indicates a slight improvement in absorption at higher levels of both solvents, yet the rates remain suboptimal, reflecting challenges in achieving efficient CO_2_ capture with the given solvent ratios. Nevertheless, the concentration of the averaged CO_2_ of such a sour gas composition is the lowest compared to the others.

Scenario 4 reveals that the highest concentrations of H_2_S in Figure 10g are found at the highest solvent levels, suggesting a negative impact of increased solvent concentrations on H_2_S removal efficiency. It is observed that effective absorption occurs at moderate concentrations of MDEA, particularly around 42% to 44% MDEA, with PZ levels maintained at or below 5%. This region is characterized by lower H_2_S concentrations, visible in the deeper blue hues indicating values potentially below 50 ppm. As the PZ concentration increases above 5%, the effectiveness of the solvent mixture in absorbing H_2_S decreases, indicated by the gradient shifting to warmer colors and higher concentrations, likely exceeding 100 ppm. For CO_2_ in Figure 10h, a low concentration is found when MDEA is at its highest between 42% and 44% and PZ is at its lowest, around 0% to 5%. This area, indicated by the darkest blues, suggests CO_2_ concentrations potentially as low as 2% or less. Based on the contour, it has a broader area of dark blue when the PZ exceeds 5% with MDEA arranging from 40% to 45%.

In Scenario 5, the H_2_S in Figure 10i and CO_2_ in Figure 10j show that as the PZ concentration increases beyond this range, the effectiveness in absorbing H_2_S declines, with H_2_S concentrations exceeding 50 ppm. Conversely, the increase in PZ improves the CO_2_ absorption.

These contour plots highlight the complex and varied impacts of MDEA and PZ concentrations on H_2_S and CO_2_ absorption rates across different scenarios. They demonstrate that optimal solvent compositions for effective gas sweetening treatment can vary significantly, depending on the specific gas composition and operational conditions. This analysis underscores the need for the careful tuning of solvent composition ratios to improve absorption efficiencies in industrial applications.

## 3. Materials and Methods

**Figure 11:**
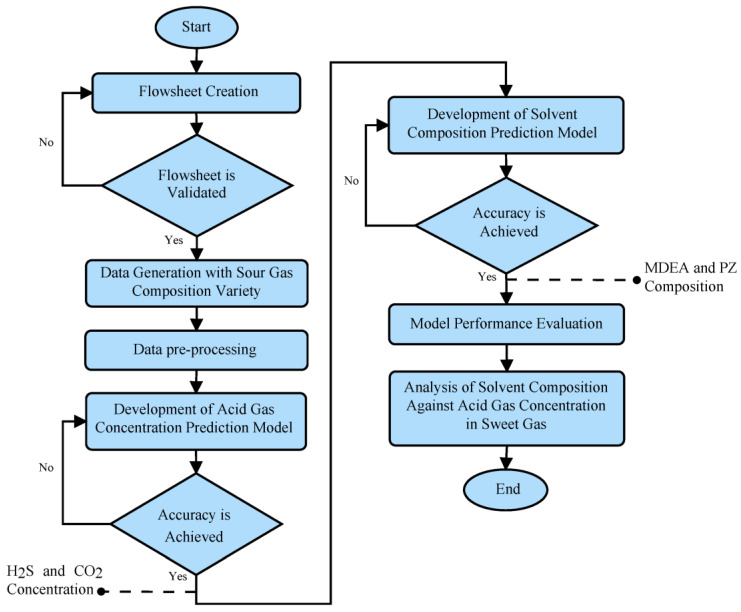
Overall research flowchart.

The framework of this study is illustrated in Figure 11. The initial step is creating a process flowsheet of AGRU using Hysys software. The validated flowsheet is further used to generate data with various sour gas compositions. Subsequently, the generated data undergo pre-processing to examine the correlation between variables.

In this study, two predictive models will be developed. The first model aims to predict the concentration of H_2_S and CO_2_. This model eliminates the complexity of simulation to predict the concentration of both acid gases. The second model predicts the solvent composition of MDEA and PZ. These models will be further compared with other comprehensive algorithms to assess the advantages of the proposed model. The developed models will be evaluated according to their performance. Lastly, the predicted concentration of H_2_S and CO_2_ together with the predicted MDEA and PZ composition are plotted to observe the effect of solvent composition on absorption performance.

### 3.1. Process Flowsheet Creation of AGRU System in Hysys

**Figure 12:**
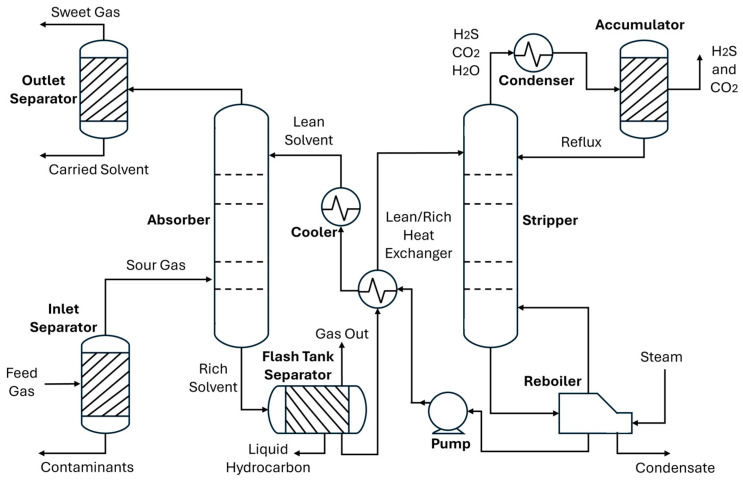
Chemical absorption-based gas sweetening flow diagram of AGRU.

The process flowsheet intends to mimic the actual operation of the AGRU system. As previously highlighted, chemical absorption has become the most widely used technique in the gas sweetening process. Figure 12 depicts the process flow diagram of a typical AGRU system. It has two main operational units, namely an absorber and stripper. The gas sweetening process is performed in an absorber column. The feed stream of sour gas initially enters a separator to remove unnecessary liquids or solids before being delivered to the absorber. The filtered sour gas further flows to the absorber from the bottom, while the solvent is fed from the top flowing counter-currently [18]. The chemical reaction between these two elements is represented in Equation (1) for H_2_S and Equation (2) for CO_2_ removals.

2RNH_2_ + H_2_S ⇌ (RNH_3_)S (1)

2RNH_2_ + CO_2_ ⇌ (RNH_2_)_2_ HCO_3_ (2)

where R in mono-, di-, or tri-ethanol; N, H, S, C, and O indicate nitrogen, hydrogen, sulfur, carbon, and oxygen, respectively. The reaction is reversible under endothermic conditions.

The cleaned gas or sweet gas enters the absorber from the top of the absorber column, then is further processed to the outlet separator, ensuring the sweet gas is free from the entrained solvent. On the other hand, the absorbed acid gas in rich solvent leaves the absorber from the bottom side to the stripper unit [19,20].

The rich solvent then flows to the flash tank separator to remove unwanted liquid hydrocarbon. It further undergoes a reheating process before entering the stripper column. Since the reaction of the gas sweetening process is reversible, as mentioned in Equations (1) and (2), the process can be facilitated by heating the solvent. Thus, in the stripper unit, heat is used to remove the absorbed acid gas from the rich solvent stream. Subsequently, the regenerated lean solvent solution is circulated back to the absorber unit for reuse in the next absorption cycle.

**Figure 13:**
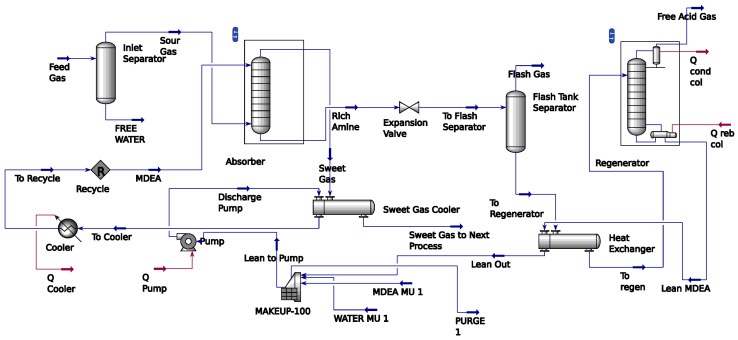
Developed flowsheet of AGRU in Hysys.

The whole AGRU system is modelled using the Hysys version 14 environment as depicted in Figure 13. This process flowsheet has been validated in previous work [12]. Employing a case study from industry from literature, this flowsheet gained acceptable accuracy with an averaged error of 2%. Thus, this flowsheet is accepted for data generation.

### 3.2. Data Generation from the Developed Process Flowsheet of AGRU

Several potential variables which influence absorption performance during the gas sweetening process are selected. These variables include sour gas properties, solvent composition, operating temperature, and absorber pressure. The sour gas properties consist of methane (CH_4_), ethane (C_2_H_6_), propane (C_3_H_8_), i-Butane (C_4_H_10_), n-Butane (C_4_H_10_), i-Pentane (C_5_H_12_), n-Pentane (C_5_H_12_), water (H_2_O), carbon dioxide (CO_2_), hydrogen sulfide (H_2_S), and nitrogen (N_2_). As a part of the investigation, the sour gas composition variety is applied to the simulation. It aims to discover the impact of sour gas composition over AGRU performance. Five scenarios of various different compositions of natural gas referred from previous studies [12,21] are chosen, as tabulated in Table 4. All properties are presented as the molar fraction.

To generate data, the Case Study tool in Aspen Hysys software is implemented. The independent variable will be iteratively simulated using a certain generation framework. Table 5 shows the framework for generating the data. For each case, it will generate 201 sample and repetitively run until the 15th case. Since there are five different sour gas compositions, the total data were generated and utilized for model development representing 15,075 samples. The generated data were further split 70:30 for the training and testing dataset. The upper limit and lower limit of MDEA composition are 35.50 wt% and 45.50 wt%, with a base value of 40.50 wt%. For PZ, the upper and lower limit are 0 wt% and 10 wt%, respectively, with a basis of 5 wt%. The temperature ranges from 38.50 °C to 71.50 °C, with the basis of 55 °C. The pressure of the absorber ranges from 37.90 bar to 77 bar, with a base value of 57.45 bar. The variables of MDEA and PZ concentrations, temperature, and pressure will be utilized to predict the concentration of H_2_S as the first predictive model. The second predictive model, solvent composition prediction, will utilize the concentration of H_2_S and CO_2_, temperature, and pressure as independent variables to predict the composition of MDEA and PZ.

### 3.3. Model Development Using XGBoost

Extreme Gradient Boosting (XGBoost) is a robust machine-learning algorithm renowned for its utility in supervised learning tasks such as classification and regression. The fundamental principle of XGBoost involves sequentially constructing an ensemble of weak models, primarily decision trees, where each successive model addresses the errors of its predecessors [22]. XGBoost integrates several enhancements to boost performance, including parallel processing for rapid tree construction, depth-first tree pruning, and internal management of missing data. It also features regularization (both L1 and L2) to handle overfitting, thereby enhancing the model’s effectiveness on new data. The algorithm’s versatility is further amplified by its ability to accommodate custom optimization objectives and evaluation metrics, making it suitable for a broad array of challenges [23]. This blend of precision, speed, and adaptability has made XGBoost a favored choice in both competitive machine learning and practical applications [24].

**Figure 14:**
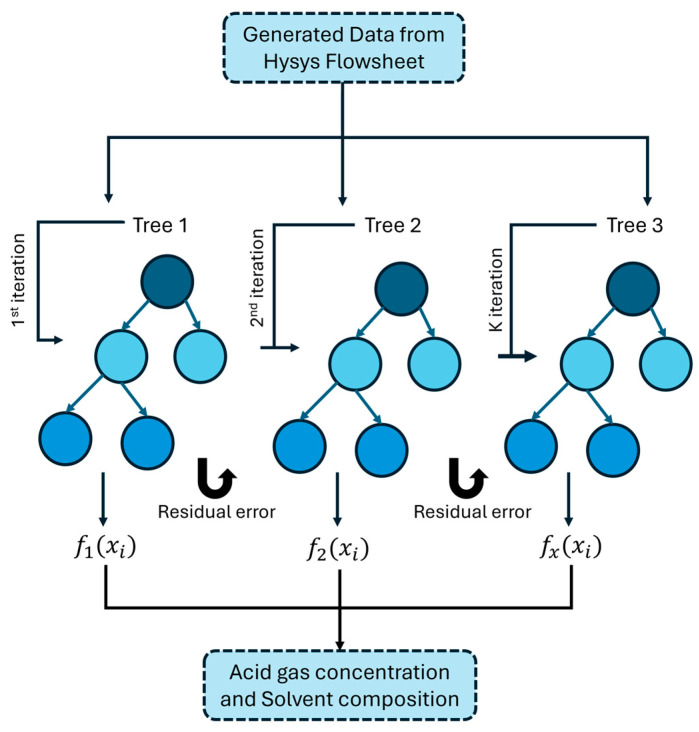
XGBoost regression framework.

In this research, XGBoost regression is utilized to estimate the concentrations of H_2_S and CO_2_ as well as the composition of the solvent mixture. XGBoost achieves this by modeling continuous output values through the interrelationships among features. It fits numerous decision trees sequentially, each aimed at refining the predictions by correcting the preceding residuals or errors, optimizing a loss function that quantifies the discrepancy between the predicted and actual values. The architecture of XGBoost is illustrated in Figure 14.

The conceptual basis of XG-Boost originates from the boosting method, represented by Equation (3):

(3) $${\hat{y_{i}}}^{k}={\hat{y_{i}}}^{k-1}+f_{k}(x_{i})$$

Here, ${\hat{y_{i}}}^{k}$ denotes the predicted output for the $i_{th}$ data point at iteration k, with $f_{k}(x_{i})$ being the estimator that enhances the previous prediction ${\hat{y_{i}}}^{k-1}$.

To prevent overfitting and optimize performance, XG-Boost introduces a regularization function into its objective or loss function for regression issues, shown in Equation (4):

(4) $$J=\sum_{i=0}^{n}L\left(y_{i},\;{\hat{y}}_{i}\right)+\sum_{k=0}^{n}\Omega (f_{k})$$

Here, n is the number of training samples, and $\Omega (f_{k})$ represents the regularization function, detailed in Equation (5):

(5) $$\Omega \left(f_{k}\right)=\gamma T+0.5\lambda \;\sum_{j=0}^{T}w_{j}^{2}$$

In this equation, T denotes the number of leaf nodes, w represents the weight of the leaves, and γ and λ are tunable hyperparameters that enhance model performance and prediction accuracy. The training proceeds iteratively, with new trees added to address the residuals of previous ones, cumulatively refining the overall prediction.

### 3.4. Model Performance Evaluation Metric

The model will be evaluated numerically through several performance metrics. The evaluation is based on three performance parameters: R-squared (R^2^), Mean Absolute Error (MAE), and Root Mean Squared Error (RMSE), as represented by Equations (6)–(8), where $y_{i}$ is the actual data, $\hat{y}$ is the predicted values, $\bar{y}$ is the mean of the actual data, and n is the number of observed data. The predictive model is accepted when the R^2^ achieves 0.9 and both of the errors are less than 1, ensuring a high prediction performance.

(6) $$R^{2}=1-\frac{\sum_{i}{\left(y_{i}-{\hat{y}}_{i}\right)}^{2}}{\sum_{i}{\left(y_{i}-{\bar{y}}_{i}\right)}^{2}}$$

(7) $$MAE=\frac{1}{n}\sum_{i=1\;}^{n}\left|y_{i}-{\hat{y}}_{i}\right|\;$$

(8) $$RMSE=\sqrt{\frac{1}{n}\sum_{i=1\;}^{n}{\left(y_{i}-{\hat{y}}_{i}\right)}^{2}}\;$$

## 4. Conclusions

This paper presents predictive techniques to predict the solvent composition for absorption-based AGRUs using MDEA and PZ as the solvent. In this paper, a predictive model of H_2_S and CO_2_ concentration has been developed. The predicted solvent composition and acid gas concentration are then evaluated to observe the behavior of absorption performance within different sour gas compositions.

Five different sour gas composition are observed. Scenario 4 produces the highest H_2_S with a peak value of 121.8 ppm and the highest CO_2_ peaking at 8.44%, while the lowest concentration of H_2_S and CO_2_ is found at Scenario 2. This resultant amount of H_2_S and CO_2_ in sweet gas is highly correlated with the molar fraction of such variables in sour gas.

The proposed model of XGBoost successfully predicts the solvent composition of MDEA and PZ, as well as the concentration of H_2_S and CO_2_. An outstanding accuracy as represented by an R^2^ of more than 0.99 is achieved at most of the tested scenarios. Compared with other comprehensive algorithms such as Linear Regression, k-Nearest Neighbors, and Support Vector Machine, the proposed XGBoost exhibits a reliable result with consistent accuracy. Thus, it can be concluded that the proposed model is robust for such application.

Finally, based on the contour plot, the composition of MDEA and PZ highly influences the absorption performance of H_2_S and CO_2_. In most scenarios, a higher MDEA composition leads to more effective absorption of H_2_S, and a higher PZ composition helps to improve the absorption of CO_2_. Both developed models will be useful to determine the suitable solvent composition for optimized absorption-based AGRUs with MDEA and PZ as the selected solvent.

## Figures and Tables

**Table 1:** Statistics of the simulated data.

Concentration in Sweet Gas	Statistic	Scenario 1	Scenario 2	Scenario 3	Scenario 4	Scenario 5
H_2_S (ppm)	Max	104.2	35.78	101.2	121.8	66.62
	Min	35.98	12.05	31.71	40.39	18.99
	Avg	53.05	18.20	43.06	66.98	33.90
CO_2_ (%)	Max	5.97	5.34	3.38	8.44	7.23
	Min	0.73	0.65	0.46	0.97	0.84
	Avg	1.31	1.16	0.84	1.71	1.51

**Table 2:** Performance metrics of the prediction for H_2_S and CO_2_ concentration.

Target	Model	Scenario	Training			Testing		
			R^2^	RMSE	MAE	R^2^	RMSE	MAE
H_2_S	XGBoost	1	0.999	0.922	0.063	0.997	0.508	0.242
		2	0.999	0.031	0.021	0.998	0.154	0.081
		3	0.999	0.087	0.055	0.998	0.455	0.205
		4	0.999	0.119	0.084	0.995	0.623	0.308
		5	0.999	0.060	0.043	0.992	0.532	0.172
	LR	1	0.563	6.168	4.846	0.508	6.094	4.830
		2	0.601	2.296	1.804	0.555	2.267	1.793
		3	0.724	5.863	4.461	0.704	5.734	4.403
		4	0.415	7.549	5.890	0.392	7.300	5.691
		5	0.562	4.118	3.231	0.592	3.976	3.136
	kNN	1	0.995	0.650	0.153	0.995	0.559	0.179
		2	0.996	0.213	0.055	0.997	0.185	0.064
		3	0.996	0.686	0.129	0.996	0.589	0.164
		4	0.989	1.010	0.226	0.983	1.205	0.295
		5	0.994	0.481	0.109	0.991	0.551	0.137
	SVM	1	0.979	1.341	0.359	0.977	1.309	0.371
		2	0.987	0.412	0.142	0.987	0.383	0.142
		3	0.992	0.952	0.222	0.996	0.597	0.214
		4	0.931	2.597	0.523	0.915	2.732	0.562
		5	0.974	0.996	0.228	0.963	1.101	0.254
CO_2_	XGBoost	1	0.999	0.003	0.002	0.994	0.038	0.011
		2	0.999	0.003	0.002	0.995	0.028	0.008
		3	0.999	0.002	0.001	0.995	0.019	0.006
		4	0.999	0.004	0.003	0.992	0.064	0.016
		5	0.999	0.004	0.002	0.991	0.059	0.013
	LR	1	0.410	0.389	0.207	0.454	0.369	0.200
		2	0.428	0.338	0.180	0.465	0.327	0.175
		3	0.468	0.203	0.113	0.511	0.192	0.112
		4	0.391	0.561	0.297	0.439	0.543	0.296
		5	0.403	0.487	0.257	0.453	0.456	0.253
	kNN	1	0.949	0.113	0.015	0.923	0.138	0.019
		2	0.953	0.096	0.013	0.922	0.125	0.017
		3	0.962	0.055	0.007	0.955	0.059	0.008
		4	0.960	0.143	0.021	0.919	0.205	0.031
		5	0.952	0.138	0.019	0.939	0.152	0.022
	SVM	1	0.878	0.176	0.007	0.876	0.175	0.071
		2	0.887	0.149	0.069	0.879	0.155	0.070
		3	0.904	0.086	0.059	0.909	0.083	0.058
		4	0.873	0.256	0.076	0.870	0.261	0.079
		5	0.873	0.225	0.079	0.883	0.211	0.078

**Table 3:** Performance metrics of prediction for MDEA and PZ concentrations.

Target	Model	Scenario	Training			Testing		
			R^2^	RMSE	MAE	R^2^	RMSE	MAE
MDEA	XGBoost	1	0.993	0.174	0.066	0.931	0.543	0.274
		2	0.991	0.193	0.081	0.905	0.638	0.244
		3	0.994	0.160	0.107	0.896	0.666	0.300
		4	0.994	0.159	0.045	0.953	0.448	0.135
		5	0.991	0.200	0.066	0.921	0.583	0.198
	LR	1	0.351	1.721	1.336	0.355	1.663	1.292
		2	0.342	1.733	1.351	0.534	1.682	1.309
		3	0.352	1.720	1.366	0.332	1.692	1.331
		4	0.443	1.594	1.216	0.462	1.518	1.167
		5	0.384	1.677	1.287	0.398	1.607	1.240
	kNN	1	0.949	0.480	0.160	0.901	0.650	0.229
		2	0.935	0.541	0.200	0.867	0.752	0.311
		3	0.905	0.658	0.341	0.814	0.893	0.526
		4	0.965	0.396	0.094	0.948	0.470	0.127
		5	0.951	0.469	0.137	0.928	0.555	0.175
	SVM	1	0.856	0.810	0.482	0.842	0.822	0.502
		2	0.826	0.891	0.556	0.808	0.905	0.579
		3	0.711	1.147	0.601	0.679	1.170	0.635
		4	0.891	0.703	0.288	0.888	0.691	0.281
		5	0.857	0.806	0.393	0.841	0.823	0.410
PZ	XGBoost	1	0.999	0.030	0.017	0.997	0.105	0.051
		2	0.999	0.035	0.020	0.997	0.114	0.050
		3	0.999	0.024	0.015	0.998	0.078	0.047
		4	0.999	0.035	0.010	0.997	0.105	0.054
		5	0.999	0.041	0.019	0.996	0.136	0.057
	LR	1	0.759	1.046	0.729	0.766	1.009	0.702
		2	0.794	0.966	0.668	0.797	0.938	0.645
		3	0.908	0.643	0.448	0.913	0.614	0.422
		4	0.617	1.314	0.964	0.651	1.232	0.919
		5	0.689	1.187	0.843	0.715	1.113	0.802
	kNN	1	0.998	0.090	0.029	0.996	0.128	0.042
		2	0.998	0.090	0.029	0.996	0.115	0.039
		3	0.999	0.064	0.022	0.999	0.061	0.028
		4	0.997	0.107	0.034	0.996	0.130	0.048
		5	0.997	0.097	0.032	0.996	0.117	0.042
	SVM	1	0.992	0.188	0.122	0.991	0.191	0.126
		2	0.993	0.177	0.114	0.993	0.174	0.118
		3	0.996	0.130	0.083	0.995	0.143	0.086
		4	0.989	0.212	0.107	0.990	0.204	0.104
		5	0.989	0.215	0.124	0.987	0.237	0.130

**Table 4:** Specification of sour gas composition.

Properties	Mol Fraction				
	Scenario 1	Scenario 2	Scenario 3	Scenario 4	Scenario 5
Methane	0.7903	0.7274	0.8294	0.7515	0.7875
Ethane	0.0646	0.1089	0.0632	0.0864	0.0318
Propane	0.0226	0.0498	0.0266	0.0219	0.0464
i-Butane	0.0122	0.0112	0.0067	0.0070	0.0081
n-Butane	0.0141	0.0157	0.0079	0.0074	0.0158
i-Pentane	0.0032	0.0047	0.0034	0.0045	0.0032
n-Pentane	0.0055	0.0050	0.0056	0.0033	0.0055
Water	0.0004	0.0008	0.0004	0.0019	0.0020
Carbon Dioxide	0.0722	0.0651	0.0416	0.1013	0.0873
Hydrogen Sulfide	0.0078	0.0025	0.0057	0.0087	0.0049
Nitrogen	0.0072	0.0089	0.0094	0.0061	0.0075
Total	1.0000	1.0000	1.0000	1.0000	1.0000

**Table 5:** Framework of data generation in Hysys.

Case Study	MDEA (%)	PZ (%)	Temperature (°C)	Pressure (bar)
1	35.50–45.50	Base	Base	Base
2	Base	0–10	Base	Base
3	Base	Base	38.50–71.50	Base
4	Base	Base	Base	37.30–70.32
5	35.50–45.50	0–10	Base	Base
6	Base	0–10	38.50–71.50	Base
7	Base	Base	38.50–71.50	37.30–70.32
8	35.50–45.50	Base	38.50–71.50	Base
9	35.50–45.50	Base	Base	37.30–70.32
10	Base	0–10	38.50–71.50	Base
11	Base	0–10	Base	37.30–70.32
12	35.50–45.50	0–10	38.50–71.50	Base
13	35.50–45.50	0–10	Base	37.30–70.32
14	Base	0–10	38.50–71.50	37.30–70.32
15	35.50–45.50	0–10	38.50–71.50	37.30–70.32

## Data Availability

Data are contained within the article.
